# Unsupervised Quantum Gate Control for Gate-Model Quantum Computers

**DOI:** 10.1038/s41598-020-67018-1

**Published:** 2020-07-01

**Authors:** Laszlo Gyongyosi

**Affiliations:** 1 School of Electronics and Computer Science, University of Southampton, Southampton, SO17 1BJ UK; 2 Department of Networked Systems and Services, Budapest University of Technology and Economics, Budapest, H-1117 Hungary; 3 MTA-BME Information Systems Research Group, Hungarian Academy of Sciences, Budapest, H-1051 Hungary

**Keywords:** Mathematics and computing, Computer science, Pure mathematics, Quantum information

## Abstract

In near-term quantum computers, the operations are realized by unitary quantum gates. The precise and stable working mechanism of quantum gates is essential for the implementation of any complex quantum computations. Here, we define a method for the unsupervised control of quantum gates in near-term quantum computers. We model a scenario in which a tensor product structure of non-stable quantum gates is not controllable in terms of control theory. We prove that the non-stable quantum gate becomes controllable via a machine learning method if the quantum gates formulate an entangled gate structure.

## Introduction

Quantum computers^1–12^ utilize quantum mechanics to solve computational problems more efficiently than traditional computers^1,3,4,13–46^. The experimental realizations of quantum computers have already begun to be implemented^1–4,8,9^ to demonstrate the quantum supremacy^3,4^ (advantages) of quantum computations^2,5–7,47^. In a gate-model quantum computer, the operations are performed by quantum gates (unitary operators)^13–15,18,20,32–36,39^. The precise and stable working mechanism of quantum gates is essential for the realization of any complex quantum computations. In particular, the quantum gates can communicate through a quantum bus^39,48–51^ that consists of the quantum systems outputted by the quantum gates, along with several auxiliary quantum systems and measurement operators. The aim of the quantum bus is analogous to that of traditional communication buses, though in a quantum computer, the quantum bus can also entangle the quantum gates. Practically, the creation of the entangled gate structure is achievable by an appropriate measurement operator applied to the auxiliary systems on the quantum bus, where the auxiliary systems are correlated with the gate outputs. Here, we are focusing on the problem of controlling non-stable quantum gates in an entangled gate structure in gate-model quantum computers. Another important aspect is the application of gate-model quantum computations^24–31,52–55^ in the near-term quantum devices of the quantum Internet^20,52,56–105^ (such as in quantum repeaters^56–58,106–114^, or in small scale quantum devices of the quantum Internet^20,56–58,65,66^).

Machine learning is about statistical inference of measured data. The aim of machine learning is to derive system models and effective control functions from noisy data (measurements)^24,115–117^. The field of machine learning is at the intersection of computer science, mathematics, artificial intelligence, and statistical sciences. Machine learning–based solutions have been applied successfully in academic and industrial studies as well as technology and marketing applications^24,115–117^. This has been possible mainly because the large amounts of data required by machine learning methods are now available due to the technical advancements in current telecommunication networks and data processing tools. The motivation behind the application of machine learning–based models for controlling complex physical systems^24,25,53,115,118–124^. is that these models can extract precise system models and optimal control functions in scenarios in which this task is hard or impossible by other control methods^24,33,53,115–117^. In a general architecture of machine learning–based controlling, the system model consists of a complex physical system subject to controlling, noisy measurement results from the physical system, a cost function, and the machine learning control unit, which outputs a control function to calibrate the physical system^24,115–117^. The machine learning unit uses the noisy measurements and the cost function as feedback information, formulating a learning loop. The aim of a machine learning–based control is to find an optimal control function that minimizes the cost function for the system calibration.

Here we define a framework for controlling entangled quantum gates in quantum computer architectures via unsupervised learning. In the system model, the quantum gates output quantum systems to a quantum bus that correlates these output quantum systems with some auxiliary quantum system (probe beams^39,48–51^). These auxiliary systems are then measured, providing information about the gate unitaries for the machine learning module. The quantum bus also contains a measurement operator to entangle the output quantum systems, which results in an entangled gate structure. In the control problem, it is assumed that a particular quantum gate oscillates randomly, i.e., a quantum gate is non-stable. We prove that this random oscillation is not controllable in terms of control theory^115–117^ if the quantum gates formulate a product system (i.e., the quantum gates are unentangled). On the other hand, the non-stable quantum gate is controllable if the quantum gates are entangled. In this case, the randomly oscillating quantum gate can be controlled by a stable quantum gate in the joint gate structure. We also define a post-processing unit that processes the measurement results, which are then fed into the machine learning control (${\mathscr{C}}$) unit to learn an optimal control function in an unsupervised manner. The ${\mathscr{C}}$ unit achieves the quantum gate calibration via the determination of an appropriate control function that minimizes a particular cost function defined for the mechanism. The output of the ${\mathscr{C}}$ block is a control parameter that achieves the calibration of the non-stable gate in the entangled gate structure. We also define a scheme for the calibration of the quantum bus states directly on the quantum bus. We provide the results for a particular gate pair in the entangled structure, but the results can be extended for an arbitrary setting with an arbitrary number of gates. The proposed framework provides an easily implementable and effective control mechanism for quantum gate structures. The results are particularly convenient for experimental quantum computers and the near-term quantum devices of the quantum Internet.

The novel contributions of our manuscript are as follows:

1. We prove that random oscillation of unitary quantum gates is not controllable in terms of control theory if the quantum gates formulate a product system. We show that the quantum gate becomes controllable if the quantum gates are entangled.
2. We show that for an entangled quantum gate structure, the randomly oscillating quantum gate can be controlled by a stable quantum gate in the entangled gate structure.
3. We define a post-processing unit that processes the measurement results, which are then fed into the machine learning unit to learn an optimal control function in an unsupervised manner.
4. The control achieves the quantum gate calibration via the determination of an appropriate control function that minimizes a particular cost function defined for the mechanism. The output of the control block is a control parameter that achieves the calibration of the non-stable gate in the entangled gate structure.
5. We define a scheme for the calibration of the quantum bus states directly on the quantum bus.
6. The proposed framework provides an effective control mechanism for entangled quantum gate structures. Since the results are independent from the level of scaling, the method is particularly convenient for experimental quantum computations and near-term quantum devices of the quantum Internet.

This paper is organized as follows. In Section 2, the related works are summarized. In Section 3, the system model is defined. Section 4 proposes the control problem. In Section 5, the control method is defined. Finally, Section 6 concludes the paper. Supplemental material is included in the Appendix.

## Related works

The related works are as follows.

The theoretical background of the gate-model quantum computer environment utilized in our manuscript can be found in^13^ and^14^.

In^14^, the authors studied the subject of objective function evaluation of computational problems fed into a gate-model quantum computer environment. The work focuses on a qubit architectures with a fixed hardware structure in the physical layout.

A scheme for the evaluation of objective function connectivity (computational pathway) in gate-model quantum computers has been proposed in^21^. Objective function examples can be found in^10,11^. A method for the optimization of the measurement procedure in gate-model quantum computers has been defined in^72^. An approach for the stabilization of the state of the quantum computer in an optimal state has been discussed in^74^. A framework for the design of quantum circuits for gate-model quantum computers has been defined in^75^. A method for the optimization of quantum memory units via quantum machine learning in near-term quantum devices has been defined in^67^.

A control method of coupled spin dynamics and the design of NMR pulse sequences by gradient ascent algorithms has been conceived in^125^.

An optimization algorithm related to gate-model quantum computer architectures is defined in^13^. In^126,127^, the authors studied some relevant attributes of the algorithm. An application of the optimization algorithm to a bounded occurrence constraint problem can be found in^18^.

In^40^, the authors studied the objective function value distributions of the optimization algorithm. In^41–43^, the authors analyzed the experimental implementation of the algorithm on near-term gate-model quantum devices.

An approximate approximation on a quantum annealer has been studied in^128^. In^129^, the authors concealed approximate quantum adders with genetic algorithms, and analyzed the experimental scenarios. The proposed model employs a machine learning algorithm via genetic algorithms, for optimizing a quantum circuit in terms of the best gate sequence to be applied to achieve a certain global unitary operation.

In^15^, the authors defined a gate-model quantum neural network model. A training method has been proposed in^73^.

For a review on the noisy intermediate-scale quantum (NISQ) era, we suggest^1^. On the subject of quantum computational supremacy, see^4^. The complexity-theoretic foundations of quantum supremacy is studied in^3^. A survey on quantum channels can be found in^24^, while for a survey on quantum computing, see^130^.

## Problem statement and system model

### Problem statement

The problems to be solved are given in Problems 1–4.

#### **Problem 1**

*Find a method to extract the gate unitary operations for the machine learning–based control of the quantum gates of the quantum computer*.

#### **Problem 2**

*Define a method to fix random oscillations of quantum gates in an entangled gate structure*.

#### **Problem 3**

*Find a P post-processing that yields the estimations of the unitary operations of the quantum gates in an arbitrary gate-model quantum computer*.

#### **Problem 4**

*Define a* ${\mathscr{C}}$ *unsupervised control method for the entangled gate structure of the quantum computer*.

The resolutions to Problems 1–4 are proposed in the Theorems and Lemmas.

### System model

The system model consist of *n* unitary gates, *U*_*i*_, *i* = 1, …, *n*, that output quantum states $|{\varphi }_{i}\rangle$, which are placed onto the *Q*_*b*_ quantum bus^39,48–51^. For simplicity, we assume qubit systems; therefore, the quantum systems are *d* = 2 dimensional, and *Q*_*b*_ is a qubus. In the *Q*_*b*_ qubus, for each $|{\varphi }_{i}\rangle$ an auxiliary qubit system ${|0\rangle }_{i}$ is associated via a CNOT gate. In a physical layer representation, the probe beam is a continuous quantum variable, i.e., a collection of a large number of photons implementable by laser or microwave pulses^39,48–51^). The CNOT gate refers to the interaction between the output states and the auxiliary systems.

To identify the correctness of the quantum gates, for each *U*_*i*_, a reference angle ±*θ*_*i*_ is associated in the phase space (i.e., the phase of ${|0\rangle }_{i}$ is rotated in the ${\mathscr{S}}$ phase space by an angle ±*θ*_*i*_). The actual phase space angle of *U*_*i*_ is identified through the measurement of ${|0\rangle }_{i}$ via the *M* homodyne measurement^39,131^. The measurement results are post-processed by *P* post-processing unit and then fed into the ${\mathscr{C}}$ machine learning control block that achieves the calibration of the *U*_*i*_ quantum gates.

Finally, the system model contains an operator *U*_*C*_ for the direct correction of the actual $|{\varphi }_{i}\rangle$ states on *Q*_*b*_ and a second homodyne measurement *M*_*b*_ that creates entanglement between the calibrated states via the measurement of the (*n* + 1)-th beam^39,131^ ${|0\rangle }_{b}$.

The aim of the machine learning–based gate controlling procedure is to calibrate the working mechanism of the quantum operations via the derivation of a *C*(·) control function. The *C*(·) control function requires the construction of a system model by ${\mathscr{C}}$ from measurement information provided by an *M* measurement phase. The measurement information is post-processed via a *P* post-processing phase and are then fed into the ${\mathscr{C}}$ procedure to determine the optimal control function.

Without loss of generality, as vector *M* refers to the measurement results, then the output of ${\mathscr{C}}$ is defined as

1 $$\partial =C(P(M)),$$

where ∂ is the control parameter. The *s* = *P*(*M*) system state is associated with a cost function *f*_*s*_, while the ∂ control parameter is associated with a cost function *f*_∂_. The functions *f*_*s*_ and *f*_∂_ formulate the *f*_*C*_ cost function subject to a minimization as

2 $${f}_{C}={f}_{s}+\gamma {f}_{\partial },$$

via the determination of an optimal control function C^*^(*s*) as

3 $${C}^{\ast }(s)=\mathop{{\rm{\arg }}\,{\rm{\min }}}\limits_{\forall C(s)}{f}_{C}(s).$$

#### Identify the unstable and stable gates

The unstable and stable unitaries of the gate-model quantum computer can be identified via a homodyne measurement applied on the auxiliary quantum states of the quantum bus. The measurement extracts relevant information about the quantum gates to determine the stable and unstable unitaries. The method and results are given by Theorem 1.

##### **Theorem 1**

*(Identify the unstable and stable gates). The unitary operators of the n quantum gates U*_*i*_, *i* = 1, …, *n of the quantum computer can be extracted via a M homodyne measurement of n auxiliary quantum systems* ${|0\rangle }_{i}$ *of Q*_*b*_.

**Proof**. Let $|0{\rangle }^{\otimes n}={\{|0\rangle }_{1},\ldots ,|0{\rangle }_{n}\}$ be an auxiliary system measured by a homodyne measurement *M*. This measurement serves for the identification of the imperfections of the *U*_*i*_, *i* = 1, …, *n* gates via the $|{\varphi }_{i}\rangle$ output systems.

In the system model, each ${|0\rangle }_{i}$ auxiliary system is physically realized by a probe beam (e.g., laser or microwave pulse). A particular *i*-th probe beam is, in fact, a continuous-variable that contains a large number of photons, each of which interacts with the *i*-th output state, $|{\varphi }_{i}\rangle$ (logically, this interaction is represented by the CNOT gate between an *i*-th pair, $|{\varphi }_{i}\rangle$ and ${|0\rangle }_{i}$). The *n* probe beams are then measured by the *M* homodyne measurement block^39,131^, such that *n* continuous variables are generated on its output.

Without loss of generality, the interaction for an *i*-th qubit can be described by the effective form of cross-Kerr nonlinearity^39,48–51^ via the ${H}_{\mathrm{int}}^{i}$ interaction Hamiltonian as

4 $${H}_{\mathrm{int}}^{i}=\hslash {\chi }_{i}{\sigma }_{Z}{a}^{\dagger }a,$$

where *χ*_*i*_ is the strength of the nonlinear interaction, *a* and ${a}^{\dagger }$ are the creation and annihilation operators^39,48–51^, and *σ*_*z*_ is the Pauli *Z*-operator.

Assuming that the interaction holds for a time ${t}_{\mathrm{int}}^{i}$ for an *i*-th probe beam ${|0\rangle }_{i}$, the interaction causes a rotation in the phase space ${\mathscr{S}}$ by a particular angle $\pm {\theta }_{{\mathscr{i}}}^{{\prime} }$ (i.e., phase shift of ${|0\rangle }_{i}$), which is defined as

5 $${\theta }_{{\mathscr{i}}}^{{\prime} }={\chi }_{i}{t}_{\mathrm{int}}^{i}.$$

Then, by $|{\varphi }_{i}\rangle$ interacting with the *i*-th probe beam, the ${\theta {\prime} }_{i}$ angle is as follows:

6 $${\theta }_{{\mathscr{i}}}^{{\prime} }=\{\begin{array}{l}{\theta }_{i},{\rm{if}}|{\varphi }_{i}\rangle ={U}_{i}|0\rangle \\ -{\theta }_{i},{\rm{if}}|{\varphi }_{i}\rangle ={U}_{i}|1\rangle \\ {\theta }_{i}+{\Delta }_{i},{\rm{if}}|{\varphi }_{i}\rangle ={V}_{i}|0\rangle \\ -({\theta }_{i}+{\lambda }_{i}),{\rm{if}}|{\varphi }_{i}\rangle ={Q}_{i}|1\rangle \end{array}\mathrm{}.$$

A detailed description of (6) is as follows: The *i*-th probe beam picks up a *θ*_*i*_ phase shift if $|{\varphi }_{i}\rangle$ is in the state ${U}_{i}|0\rangle$ and a −*θ*_*i*_ phase shift if $|{\varphi }_{i}\rangle$ is in the state *U*_*i*_|1〉, where *U*_*i*_ is the transformation associated with the *i*-th unitary gate. Hence, for each *U*_*i*_, a reference angle *θ*_*i*_ exists that identifies the correct working mechanism of gate *U*_*i*_. On the other hand, if $|{\varphi }_{i}\rangle$ is not in the state *U*_*i*_|0〉—i.e., the *i*-th gate realizes the unitary *V*_*i*_ with output $|{\varphi }_{i}\rangle ={V}_{i}|0\rangle$, where *V*_*i*_ ≠ *U*_*i*_—then the *i*-th probe beam has a phase shift ${\theta }_{{\mathscr{i}}}^{{\prime} }$ as

7 $${\theta }_{{\mathscr{i}}}^{{\prime} }={\theta }_{i}+{\varDelta }_{i}.$$

Specifically, if the *i*-th gate realizes the unitary *Q*_*i*_, so $|{\varphi }_{i}\rangle$ is not in the state *U*_*i*_|1〉, i.e., $|{\varphi }_{i}\rangle ={Q}_{i}|1\rangle$, where *Q*_*i*_ ≠ *U*_*i*_, then the phase shift is as

8 $$-{\theta }_{{\mathscr{i}}}^{{\prime} }=-\,({\theta }_{i}+{\lambda }_{i}).$$

The *θ*_*i*_ reference values are known in the model because the ideal gate unitaries are also known; therefore, the errors Δ_*i*_ and *λ*_*i*_ can be determined as follows: Since a coherent state in the phase space is associated with an *x* position and a *p* momentum parameter, the *M* homodyne measurement is in fact a projection of the elements of ${\mathscr{S}}$ onto the *x*-axis^39,131^. Therefore, from the $M({|\tilde{0}\rangle }_{i})$ projection, where ${|\tilde{0}\rangle }_{i}$ is the probe beam state after the interaction, the phase shift of the *i*-th probe beam is evaluated as

9 $$M({|\tilde{0}\rangle }_{i})=\{\begin{array}{l}x\,\cos ({\theta }_{i}),{\rm{if}}|{\varphi }_{i}\rangle ={U}_{i}|0\rangle \\ x\,\cos (-{\theta }_{i}),{\rm{if}}|{\varphi }_{i}\rangle ={U}_{i}|1\rangle \\ x\,\cos ({\theta }_{i}+{\Delta }_{i})\,{\rm{if}}|{\varphi }_{i}\rangle ={V}_{i}|0\rangle \\ x\,\cos (-{\theta }_{i}-{\lambda }_{i})\,{\rm{if}}|{\varphi }_{i}\rangle ={Q}_{i}|1\rangle \end{array}\mathrm{}.$$

Thus, from the result $M({|\tilde{0}\rangle }_{i})$, the quantities Δ_*i*_ and *λ*_*i*_ can be determined as

10 $${\Delta }_{i}={\cos }^{-1}\left(\frac{M({|\tilde{0}\rangle }_{i})}{x}\right)-{\theta }_{i}$$

and

11 $${\lambda }_{i}=-\left({\cos }^{-1}\left(\frac{M({|\tilde{0}\rangle }_{i})}{x}\right)+{\theta }_{i}\right),$$

where *x* and *θ*_*i*_ are known parameters. In fact, we do not have to know whether the actual state of $|{\varphi }_{i}\rangle$ is $|{\varphi }_{i}\rangle ={V}_{i}|0\rangle$ or $|{\varphi }_{i}\rangle ={Q}_{i}|1\rangle$, since only the difference between the actual angle ${\theta }_{{\mathscr{i}}}^{{\prime} }$ and the reference angle *θ*_*i*_ (see (10) and (11)) is required to establish the ${\mathscr{C}}$ block.

An *i*-th gate *U*_*i*_ can therefore be referred via the following operations in function of (10) and (11):

12 $${U}_{i}=\{\begin{array}{l}{U}_{i},\,{\rm{if}}\,{\Delta }_{i}=0\,{\rm{or}}\,{\lambda }_{i}=0\\ {V}_{i},\,{\rm{if}}\,{\Delta }_{i}\ne 0\\ {Q}_{i},\,{\rm{if}}\,{\lambda }_{i}\ne 0\end{array}\mathrm{}.$$

In the next step, the $|{\varphi }_{i}\rangle$ states are entangled by the *M*_*b*_ homodyne measurement block. For a particular pair $\{|{\varphi }_{i}\rangle ,|{\varphi }_{j}\rangle \}$, the aim of the quantum bus^39,48–51^ is to achieve the entangled system |Φ〉 = *U*_i_*U*_j_|*β*〉, where $|\beta \rangle$ is a Bell state, while *U*_*i*_ and *U*_*j*_ are the unitaries of the *i*-th and *j*-th unitary gates. After the *M*_*b*_ measurement, the operations *U*_*i*_ and *U*_*j*_ therefore formulate the entangled system *U*_*i*_*U*_*j*_, since $|{\varphi }_{i}\rangle ={U}_{i}|{\psi }_{i}\rangle$ and $|{\varphi }_{j}\rangle ={U}_{j}|{\psi }_{j}\rangle$ for some inputs $|{\psi }_{i}\rangle$ and $|{\psi }_{j}\rangle$.

The interaction Hamiltonian for the probe beam ${|0\rangle }_{b}$ is ${H}_{\mathrm{int}}=\hslash {\chi }_{b}{\sigma }_{Z}{a}^{\dagger }a$, where *χ*_*b*_ is the strength of the nonlinear interaction^39,48–51^. For a given interaction time *t*_*b*_, the interaction with a particular $|{\varphi }_{i}\rangle$ causes a rotation $\pm {\omega }_{i}^{b}$ in the angle of the probe beam ${|0\rangle }_{b}$ with angle ${\omega }_{i}^{b}={\chi }_{b}{t}_{b}$. For a corrected pair $\{|{\varphi }_{i}\rangle ,|{\varphi }_{j}\rangle \}$, the result of the ${M}_{b}({|0\rangle }_{b})$ homodyne measurement is $x\,\cos ({\omega }_{i}^{b}+{\omega }_{j}^{b})$ or $x\,\cos ({\omega }_{i}^{b}-{\omega }_{j}^{b})$. This is because for $\{|{\varphi }_{i}\rangle ,|{\varphi }_{j}\rangle \}={U}_{i}{|0\rangle }_{i}{U}_{j}{|0\rangle }_{j}$ or $\{|{\varphi }_{i}\rangle ,|{\varphi }_{j}\rangle \}={U}_{i}{|1\rangle }_{i}{U}_{j}{|1\rangle }_{j}$, the probe beam ${|0\rangle }_{b}$ is shifted by ${\omega }_{i}^{b}$ for a ${U}_{i}|0\rangle$ state and by $-{\omega }_{i}^{b}$ for a ${U}_{i}|1\rangle$ state; thus, in this case, the phase shift is ${\omega }_{i}^{b}+{\omega }_{j}^{b}$. On the other hand, if $\{|{\varphi }_{i}\rangle ,|{\varphi }_{j}\rangle \}={U}_{i}{|0\rangle }_{i}{U}_{j}{|1\rangle }_{j}$ or $\{|{\varphi }_{i}\rangle ,|{\varphi }_{j}\rangle \}={U}_{i}{|1\rangle }_{i}{U}_{j}{|0\rangle }_{j}$, the resulting phase shift is $|{\omega }_{i}^{b}-{\omega }_{j}^{b}|$, which yields $x\,\cos ({\omega }_{i}^{b}-{\omega }_{j}^{b})$. If ${M}_{b}({|0\rangle }_{b})$ results in $x\,\cos ({\omega }_{i}^{b}-{\omega }_{j}^{b})$, an auxiliary NOT gate is applied to either qubit of an *ij* pair $\{|{\varphi }_{i}\rangle ,|{\varphi }_{j}\rangle \}$.

Therefore, the unstable and stable quantum gates can be identified via *Q*_*b*_ and *M*, that concludes the proof. ■

#### NISQ applications

A straightforward NISQ^1^ application of the system model is in trapped ion scenarios or in superconducting circuits. The explicit number of gates, gate fidelities, and total error of the protocol are external parameters in the system model that depend on the actual physical-layer apparatus. The control theory behind the system model and the definition of the machine learning control unit makes implementable the results via near-term technologies in experiment. In particular, a near-term application is in qubit gate-model quantum computer architectures considering the case of a large number of qubits and quantum gates, $n\gg 1$^2,5–7^. As a future work, our aim is to analyze the performance of the system model in these scenarios.

The proposed system model utilizes a *Q*_*b*_ quantum bus, however *U*_*C*_ controlling block, the *P* post-processing block and the ${\mathscr{C}}$ machine learning control block can also be implemented in different practical scenarios. As follows, the system model is not limited for quantum buses allowing a widespread application in experiment. Other practical application scenarios of the results include measurement control problems and measurement optimization in quantum computations, qubit control and readout, objective function evaluation in gate-model quantum computes for solving optimization problems, and optimization of tasks of measurement-based quantum information processing. An aim is to extend the application of the proposed system model into these directions also.

**Figure 1:**
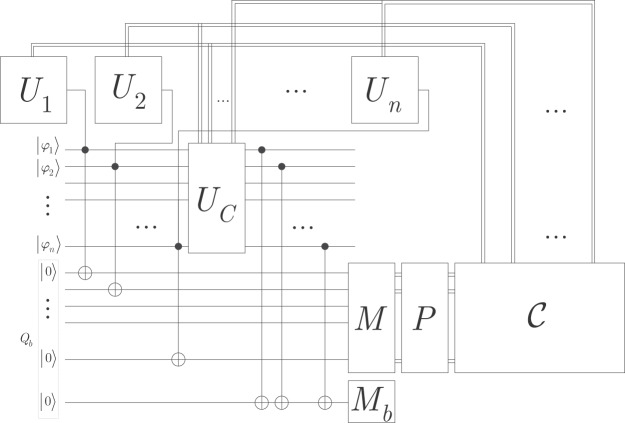
Schematic model of the unsupervised quantum gate control mechanism. The output states $|{\varphi }_{1}\rangle \ldots |{\varphi }_{n}\rangle$ of unitaries *U*_*i*_, *i* = 1, …, *n* of the quantum computer are placed onto the qubus *Q*_*b*_ (depicted by dashed frame). For each *U*_*i*_, a reference phase state angle *θ*_*i*_ is defined. For each $|{\varphi }_{i}\rangle$ an auxiliary system ${|0\rangle }_{i}$ is set using CNOT gates. The auxiliary qubits are measured by the first homodyne measurement block *M*. The measurement results (double lines refer to classical information) are processed by a *P* post-processing block, and by a ${\mathscr{C}}$ machine learning control block. Using the results of ${\mathscr{C}}$, operator *U*_*C*_ corrects of the $|{\varphi }_{i}\rangle$ states on *Q*_*b*_. The second homodyne measurement, *M*_*b*_, entangles the corrected states of *Q*_*b*_ using the probe beam ${|0\rangle }_{b}$.

The schematic model of the quantum gate controlling method is depicted in Fig. 1^47^. For simplicity, the figure shows only one-qubit unitaries, however the results hold for arbitrary quantum gates. The *U*_*C*_, *P*, and ${\mathscr{C}}$ operations are defined in Section 4.

## Quantum gate control

The aim of the ${\mathscr{C}}$ block is to achieve a machine learning–based method for controlling the unitary gates using the results of the *M* homodyne measurement block post-processed by *P*. The *U*_*C*_ operation for controlling the quantum states directly on the qubus is also defined. First, we give the problem statement in terms of control theory^115–117^.

### Method

Let us assume that for each *i*-th quantum system $|{\varphi }_{i}\rangle$, a reference angle *θ*_*i*_ is defined (see Theorem 1). This angle identifies a correct working mechanism of the unitary gate *U*_*i*_. Focusing on qubit gates, for an *i*-th gate, the reference operation *U*_*i*_ is defined as

13 $${U}_{i}=\exp (-i{H}_{i}{t}_{{U}_{i}}/\hslash )=\,\cos ({t}_{{U}_{i}}/\hslash )I-i\,\sin ({t}_{{U}_{i}}/\hslash ){H}_{{U}_{i}},$$

where ${t}_{{U}_{i}}$ is the application time of unitary *U*_*i*_, $\hslash$ is the reduced Planck constant (we set $\hslash =1$), and *I* is the identity operator, while ${H}_{{U}_{i}}$ is a Hamiltonian with energy *E*_*i*_:

14 $${E}_{i}=\frac{1}{2}\hslash 2\pi ({f}_{i}),$$

where *f*_*i*_ is the frequency:

15 $${f}_{i}=\frac{1}{{T}_{i}}$$

where *T*_*i*_ is the period time.

Focusing on a particular pair $\{|{\varphi }_{i}\rangle ,|{\varphi }_{j}\rangle \}$, the corresponding *U*_*i*_ and *U*_*j*_ reference operations are identified as follows: Let us assume that the application time of the *U*_*i*_ unitary is ${t}_{{U}_{i}}$, with period time ${\pi }_{i}={T}_{i}=z{t}_{{U}_{i}}$, *z* > 0, and for *U*_*j*_, the application time is ${t}_{{U}_{j}}$, with period time ${\pi }_{j}={T}_{j}=y{t}_{{U}_{j}}$, *y* > 0. Then, by introducing quantities *A* and *B* for *U*_*i*_, as

16 $$A=\,\cos ({t}_{{U}_{i}}),$$

17 $$B=\,\sin ({t}_{{U}_{i}}),$$

and quantities *C* and *D* for *U*_*j*_, as

18 $$C=\,\cos ({t}_{{U}_{j}}),$$

19 $$D=\,\sin ({t}_{{U}_{j}}),$$

the *U*_*i*_ and *U*_*j*_ operations can be rewritten as

20 $${U}_{i}=AI-iB{H}_{{U}_{i}},$$

and

21 $${U}_{j}=CI-iD{H}_{{U}_{j}},$$

with the corresponding Hamiltonians ${H}_{{U}_{i}}$, ${H}_{{U}_{j}}$. The related energy operators are ${E}_{i}=\frac{1}{2}\hslash 2\pi ({f}_{i})$, ${E}_{j}=\frac{1}{2}\hslash 2\pi ({f}_{j})$, where ${f}_{i}=\frac{1}{{T}_{i}}$ and ${f}_{j}=\frac{1}{{T}_{j}}$.

In the control problem, we assume a scenario in which the *U*_*i*_ gate works improperly, which leads to imperfect oscillations, while *U*_*j*_ works perfectly. The aim of the ${\mathscr{C}}$ is to achieve the stabilization of *U*_*i*_ using the fact that *M*_*b*_ creates the entangled structure *U*_*i*_*U*_*j*_, since *M*_*b*_ entangles the qubus states. The challenge here is therefore the stabilization of *U*_*i*_ such that it randomly oscillates (i.e., *U*_*i*_ is non-stable) while *U*_*j*_ is stable in *U*_*i*_*U*_*j*_. In the control problem, this random oscillation cannot be corrected by a controlling parameter applied directly to *U*_*i*_. The problem is then to find a way to correct the random oscillations by exploiting the fact that *U*_*i*_*U*_*j*_ is an entangled structure.

As we show in Theorem 2, in an entangled structure *U*_*i*_*U*_*j*_, it is possible to fix the random oscillations of *U*_*i*_ by controlling *U*_*j*_. On the other hand, if ${U}_{i}\otimes {U}_{j}$ is a product system, then calibration is not possible.

#### **Theorem 2**

*(Controlling of entangled gate structure). In an entangled gate structure U*_*i*_*U*_*j*_*, a randomly oscillating non-stable gate U*_*i*_ *is controllable via a stable gate U*_*j*_. *In a product system* ${U}_{i}\otimes {U}_{j}$, *the non-stable gate U*_*i*_ *is not controllable via the control of a stable gate U*_*j*_.

**Proof**. First, let assume that *U*_*i*_ and *U*_*j*_ are formulating the entangled structure *U*_*i*_*U*_*j*_ via the *M*_*b*_ measurement on the qubus. Using the parameters *A*, *B* of *U*_*i*_ (see (16) and (17)) and *C*, *D* of *U*_*j*_ (see (18) and (19)), the controlling problem in terms of control theory^115–117^ is as follows.

Using a Galerkin expansion and a generalized mean-field system formulation^115^ for the description of the controlling, for a non-stable gate *U*_*i*_ in the joint system *U*_*i*_*U*_*j*_, a growth rate^115–117^ parameter *μ*_*i*_ is defined as

22 $${\mu }_{i}={\beta }_{i}^{0}-{\beta }_{i}^{i}({A}^{2}+{B}^{2})-{\beta }_{i}^{j}({C}^{2}+{D}^{2}),$$

where ${\beta }_{i}^{0}$ is the initial growth rate^115–117^, ${\beta }_{i}^{i}$ is the parameter for growth-rate change of *μ*_*i*_ due to $\sqrt{({A}^{2}+{B}^{2})}$, while ${\beta }_{i}^{j}$ is the parameter for growth-rate of *μ*_*i*_ due to $\sqrt{({C}^{2}+{D}^{2})}$ for *U*_*i*_.

Then, let ${D}_{t}(x)=dx/dt$, and define sets ${{\mathscr{C}}}_{{U}_{i}}:\{{D}_{t}(A),{D}_{t}(B)\}$ and ${{\mathscr{C}}}_{{U}_{j}}:\{{D}_{t}(C),{D}_{t}(D)\}$. For *U*_*i*_, the quantity *D*_*t*_(*A*) in set ${{\mathscr{C}}}_{{U}_{i}}$ is as

23 $${D}_{t}(A)={\mu }_{i}A-{F}_{i}B,$$

where *F*_*i*_ is a parameter for the frequency defined as

24 $${F}_{i}={f}_{i}+{\delta }_{i}^{i}({A}^{2}+{B}^{2})+{\delta }_{i}^{j}({C}^{2}+{D}^{2}),$$

where *f*_*i*_ is the initial frequency, ${f}_{i}=\frac{1}{{T}_{i}}$, ${\delta }_{i}^{i}$ is a parameter for frequency-change due to term $\sqrt{({A}^{2}+{B}^{2})}$, while ${\delta }_{i}^{j}$ is a parameter for frequency-change due to term $\sqrt{({C}^{2}+{D}^{2})}$; while *D*_*t*_(*B*) in ${{\mathscr{C}}}_{{U}_{i}}$ is as

25 $${D}_{t}(B)={F}_{i}A+{\mu }_{i}B.$$

For unitary *U*_*j*_, we define *μ*_*j*_ as

26 $${\mu }_{j}={\beta }_{j}^{0}-{\beta }_{j}^{i}({A}^{2}+{B}^{2})-{\beta }_{j}^{j}({C}^{2}+{D}^{2}),$$

where ${\beta }_{j}^{0}$ is the initial growth rate, ${\beta }_{j}^{i}$ is the parameter for growth-rate of *μ*_*j*_ due to $\sqrt{({A}^{2}+{B}^{2})}$, while ${\beta }_{j}^{j}$ is the parameter for growth-rate of *μ*_*j*_ due to $\sqrt{({C}^{2}+{D}^{2})}$; and *D*_*t*_(*C*) from ${{\mathscr{C}}}_{{U}_{j}}$ is as

27 $${D}_{t}(C)={\mu }_{j}C-{F}_{j}D,$$

where *F*_*j*_ is a parameter for the frequency as

28 $${F}_{j}={f}_{j}+{\delta }_{j}^{i}({A}^{2}+{B}^{2})+{\delta }_{j}^{j}({C}^{2}+{D}^{2}),$$

where *f*_*j*_ is the initial frequency, ${f}_{j}=\frac{1}{{T}_{j}}$, ${\delta }_{j}^{i}$ is a parameter for frequency-change due to $\sqrt{({A}^{2}+{B}^{2})}$, while ${\delta }_{j}^{j}$ is a parameter for frequency-change due to $\sqrt{({C}^{2}+{D}^{2})}$; and *D*_*t*_(*D*) from ${{\mathscr{C}}}_{{U}_{j}}$ is as

29 $${D}_{t}(D)={F}_{j}C+{\mu }_{j}D+\partial ,$$

where ∂ is a control parameter.

Since for the *U*_*i*_*U*_*j*_ entangled structure, in (22) the terms ${\beta }_{i}^{i}({A}^{2}+{B}^{2})$ and ${\beta }_{i}^{j}({C}^{2}+{D}^{2})$ are non-vanishing, making the *U*_*i*_ gate to be controllable via *U*_*j*_. This connection still holds for *U*_*i*_*U*_*j*_ after some simplifications of the system model.

Let simplify the description *U*_*i*_*U*_*j*_, as follows. For *U*_*i*_, let use *F*_*i*_ = 1, and set ${\beta }_{i}^{i}={\beta }_{i}^{j}=1$ in (22). Then, by using (20) and (21), we redefine (22) as

30 $${\mu }_{i}={\beta }_{i}^{0}-({A}^{2}+{B}^{2})-({C}^{2}+{D}^{2}).$$

After the *M*_*b*_ measurement, the yielding system of *U*_*i*_ can be evaluated via (30) as

31 $${D}_{t}(A)={\mu }_{i}A-B,$$

and

32 $${D}_{t}(B)=A+{\mu }_{i}B.$$

For *U*_*j*_, set ${\beta }_{j}^{i}={\beta }_{j}^{j}=0$, thus (26) can be rewritten as

33 $${\mu }_{j}={\beta }_{j}^{0},$$

and set ${\delta }_{j}^{i}={\delta }_{j}^{j}=0$ in (28), thus (28) can be rewritten as

34 $${F}_{j}={f}_{j}=\frac{1}{{T}_{j}}.$$

Thus, (27) and (29) can be reevaluated as

35 $${D}_{t}(C)={\beta }_{j}^{0}C-{f}_{j}D,$$

and

36 $${D}_{t}(D)={\beta }_{j}^{0}D+{f}_{j}C+\partial .$$

As follows, for the simplified model (30–36) of *U*_*i*_*U*_*j*_, the *U*_*i*_ gate remains controllable via *U*_*j*_ since terms ${\beta }_{i}^{i}({A}^{2}+{B}^{2})$ and ${\beta }_{i}^{j}({C}^{2}+{D}^{2})$ are non-vanishing in (30). However, this is not the case if ${U}_{i}\otimes {U}_{j}$ formulate a product state system with unentangled gates.

The system model of the product system ${U}_{i}\otimes {U}_{j}$ is derived follows. Let assume that gates *U*_*i*_ and *U*_*j*_ are formulating a product state system ${U}_{i}\otimes {U}_{j}$. In this case, the gates *U*_*i*_ and *U*_*j*_ in ${U}_{i}\otimes {U}_{j}$ are unentangled, thus modifications required in the system model.

In terms of control theory, the ${U}_{i}\otimes {U}_{j}$ product system is analogous to a linearization around the ${A}^{2}={B}^{2}={C}^{2}={D}^{2}=0$ (fixed point of the model). This linearization breaks the entangled structure *U*_*i*_*U*_*j*_, leading to ${\beta }_{i}^{i}={\beta }_{i}^{j}={\beta }_{j}^{i}={\beta }_{j}^{j}=0$, and ${\delta }_{i}^{i}={\delta }_{i}^{j}={\delta }_{j}^{i}={\delta }_{j}^{j}=0$.

Thus, for ${U}_{i}\otimes {U}_{j}$, the terms ${\beta }_{i}^{i}({A}^{2}+{B}^{2})$ and ${\beta }_{i}^{j}({C}^{2}+{D}^{2})$ are vanishing in (22), thus (22) picks up the formula of

37 $${\mu }_{i}={\beta }_{i}^{0},$$

thus the resulting system model for ${U}_{i}\otimes {U}_{j}$ is as

38 $${D}_{t}(A)={\beta }_{i}^{0}A-{f}_{i}B,$$

39 $${D}_{t}(B)={f}_{i}A+{\beta }_{i}^{0}B.$$

40 $${D}_{t}(C)={\beta }_{j}^{0}C-{f}_{j}D,$$

and

41 $${D}_{t}(D)={f}_{j}C+{\beta }_{j}^{0}D+\partial .$$

As follows from the system model (38–41) of ${U}_{i}\otimes {U}_{j}$, for *U*_*i*_ the quantity $\sqrt{{A}^{2}+{B}^{2}}$ grows without bound (i.e., *U*_*i*_ is non-stable), while for *U*_*j*_, the quantity $\sqrt{{C}^{2}+{D}^{2}}$ converges to zero for ∂ = 0 (i.e., *U*_*j*_ is stable)^115–117^,. Thus, in terms of control theory, in system (38–41), the randomly fluctuating quantum gate *U*_*i*_ cannot controlled by *U*_*j*_. As a corollary, while *U*_*i*_ is controllable in the entangled structure *U*_*i*_*U*_*j*_, in a tensor product system ${U}_{i}\otimes {U}_{j}$ is not controllable.

The proof is concluded here. ■

### Cost function

The cost function *f*_*C*_(*U*_*i*_*U*_*j*_) for the controlling of the *U*_*i*_*U*_*j*_ entangled gate structure is a subject to a minimization, and defined as

42 $${f}_{C}({U}_{i}{U}_{j})={\tilde{f}}_{{U}_{i}}+\gamma {f}_{\partial }$$

where

43 $${\tilde{f}}_{{U}_{i}}={A}^{2}+{B}^{2}$$

and

44 $${f}_{\partial }={\partial }^{2},$$

where ∂ is the control parameter (75), while *γ* is a penalization parameter^115–117^.

Let *C*(*U*_*i*_*U*_*j*_) be a control function defined for the joint structure *U*_*i*_*U*_*j*_ as

45 $$\partial =C({U}_{i}{U}_{j}),$$

and let Pr(*A*, *B*, *C*, *D*) be the probability density associated to a current values of *A*, *B*, *C*, *D* of the entangled structure *U*_*i*_*U*_*j*_. At a particular *A*, *B* of *U*_*i*_, the result of ∂ on *U*_*i*_ can be evaluated by the ${\partial }_{{U}_{i}}={\mathbb{E}}(C({U}_{i}{U}_{j})|A,B)$ expectation value as

46 $${\partial }_{{U}_{i}}=\int \int \,Pr(A,B,C,D)C({U}_{i}{U}_{j})dCdD\mathrm{}.$$

At a given *C*, *D* of *U*_*j*_, the result of ∂ on *U*_*j*_ can be evaluated the expectation value ${\partial }_{{U}_{j}}={\mathbb{E}}(C({U}_{i}{U}_{j})|C,D)$ as

47 $${\partial }_{{U}_{j}}=\int \int \,Pr(A,B,C,D)C({U}_{i}{U}_{j})dAdB\mathrm{}.$$

The terms (45), (46) and (47) will be specified further in Section 5 via the determination of the controlling function *C*(*U*_*i*_*U*_*j*_).

### Post-processing

#### **Lemma 1**

*P post-processing on the results of the M homodyne measurement yields the estimations of the unitaries of the quantum gates*.

**Proof**. Focusing on a gate-pair {*U*_*i*_, *U*_*j*_}, the aim of the *P* post-processing method is to achieve the estimates $\tilde{A}$, $\tilde{B}$ and $\tilde{C}$, $\tilde{D}$ using the results of the *M* measurement. The estimated quantities are then fed into the ${\mathscr{C}}$ block that achieves the automated control of the quantum gates.

The quantities *A*, *B C*, and *D* are derived from the *M* measurement as follows: Let us focus on a *i*-th unitary *U*_*i*_ with quantities *A*, *B* and reference phase space angle *θ*_*i*_. The quantities Δ_*i*_ (10) and *λ*_*i*_ (11) are also computed in the *P* post-processing phase using the $M({|\tilde{0}\rangle }_{i})$ measurement (see (9)) of the *i*-th probe beam ${|\tilde{0}\rangle }_{i}$. These steps yield the estimates $\tilde{A}$ and $\tilde{B}$ as follows: Since at a particular ${t}_{{U}_{i}}$ application time of *U*_*i*_, at Δ_*i*_ = 0 or *λ*_*i*_ = 0,

48 $$\tilde{A}=\,\cos ({\varsigma }_{i}{\theta }_{i})=\,\cos ({t}_{{U}_{i}}),$$

49 $$\tilde{B}=\,\sin ({\varsigma }_{i}{\theta }_{i})=\,\sin ({t}_{{U}_{i}}),$$

where

50 $${\varsigma }_{i}=\frac{{t}_{{U}_{i}}}{{\theta }_{i}}.$$

Therefore, for Δ_*i*_ ≠ 0, the *V*_*i*_ unitary is performed instead of *U*_*i*_:

51 $$\tilde{A}=\,\cos ({\varsigma }_{i}({\theta }_{i}+{\phi }_{i}))=\,\cos ({t}_{{U}_{i}}+{t}_{{\phi }_{i}}),$$

52 $$\tilde{B}=\,\sin ({\varsigma }_{i}({\theta }_{i}+{\phi }_{i}))=\,\sin ({t}_{{U}_{i}}+{t}_{{\phi }_{i}}),$$

where

53 $${\phi }_{i}=\{\begin{array}{l}{\Delta }_{i},\,{\rm{if}}\,{\theta }_{{\mathscr{i}}}^{{\prime} } < {\theta }_{i}\\ -{\Delta }_{i},\,{\rm{if}}\,{\theta }_{{\mathscr{i}}}^{{\prime} } > {\theta }_{i}\end{array},$$

where ${\theta }_{{\mathscr{i}}}^{{\prime} }$ is given in (7), and ${t}_{{\phi }_{i}}$ is as

54 $${t}_{{\phi }_{i}}={\varsigma }_{i}({\theta }_{i}+{\phi }_{i})-{t}_{{U}_{i}}.$$

For *λ*_*i*_ ≠ 0, the *Q*_*i*_ unitary is performed instead of *U*_*i*_; thus,

55 $$\tilde{A}=\,\cos ({\varsigma }_{i}({\theta }_{i}+{\varphi }_{i}))=\,\cos ({t}_{{U}_{i}}+{t}_{{\varphi }_{i}}),$$

56 $$\tilde{B}=\,\sin ({\varsigma }_{i}({\theta }_{i}+{\varphi }_{i}))=\,\sin ({t}_{{U}_{i}}+{t}_{{\varphi }_{i}}),$$

where

57 $${\varphi }_{i}=\{\begin{array}{l}-\,{\lambda }_{i},\,{\rm{if}}\,-\,{\theta {\prime} }_{i} > -\,{\theta }_{i}\\ {\lambda }_{i},\,{\rm{if}}\,-\,{\theta {\prime} }_{i} < -\,{\theta }_{i}\end{array},$$

where $-{\theta {\prime} }_{i}$ is as given in (8) and ${t}_{{\varphi }_{i}}$ is as

58 $${t}_{{\varphi }_{i}}={\varsigma }_{i}({\theta }_{i}+{\varphi }_{i})-{t}_{{U}_{i}}.$$

These results straightforwardly follow for *U*_*j*_, with the corresponding parameters Δ_*j*_, *λ*_*j*_, ${t}_{{U}_{j}}$, reference angle *θ*_*j*_, and ${\varsigma }_{j}$. ■

The machine learning–based control of the randomly oscillating non-stable gate *U*_*i*_ in the *U*_*i*_*U*_*j*_ joint structure is discussed in Section 5.

## Unsupervised Control of Entangled Gates

### **Theorem 3**

*(Quantum gate calibration). For an entangled gate structure U*_*i*_*U*_*j*_ *with a non-stable U*_*i*_, *the* ${\mathscr{C}}$ *block calibrates the quantum gates via the optimal control function* ${C}^{\ast }({U}_{i}{U}_{j})=\sqrt{{\bar{L}}_{act}}$, *where* ${\bar{L}}_{act}$ = ∏^2^${\beta }_{i}^{0}/0.2(({\tilde{A}}^{2}+{\tilde{B}}^{2})+({\tilde{C}}^{2}+{\tilde{D}}^{2}))$, *where* ∏ *is a controlling amplitude*, *while* $\tilde{A}$, $\tilde{B}$, $\tilde{C}$, $\tilde{D}$ *are determined via P post-processing*.

**Proof**. The ${\mathscr{C}}$ block gets as input the post-processed results $P(M{(|\tilde{0}\rangle }_{i}))$, *i* = 1, …, *n*. The *C*$(\tilde{A},\tilde{B},\tilde{C},\tilde{D})$ control function of the joint system *U*_*i*_*U*_*j*_ is evaluated via the ${\mathscr{C}}$ block, where $\tilde{A}$, $\tilde{B}$, $\tilde{C}$, $\tilde{D}$ are determined by *P* post-processing step.

The ${\mathscr{C}}$ block operates via a set ${{\mathscr{S}}}_{f}$ of operations, as

59 $${{\mathscr{S}}}_{f}:\{{S}_{e},{S}_{t}\},$$

where *s*_*e*_ is a set of elementary operations *S*_*e*_ = {±, ×, /}, while *S*_*t*_ = {exp, sin, ln, tanh} is a set of transcendental functions^115–117^.

Focusing on a particular pair of unitaries {*U*_*i*_*U*_*J*_}, where *U*_*i*_ is not stable while *U*_*j*_ is a stable gate, the ${\mathscr{C}}$ block iterates until the optimal control function C^*^(*U*_*i*_*U*_*j*_) is determined. The output of the ${\mathscr{C}}$ block is the ∂^*^ optimal control parameter of the for the joint structure *U*_*i*_*U*_*j*_, as

60 $$\begin{array}{rcl}{\partial }^{\ast } & = & \int \int \,{\rm{\Pr }}(\tilde{A},\tilde{B},\tilde{C},\tilde{D}){C}^{\ast }(\tilde{A},\tilde{B},\tilde{C},\tilde{D})d\tilde{A}d\tilde{B}d\tilde{C}d\tilde{D}\\  & = & {\partial }_{1}{\partial }_{2},\end{array}$$

where $\Pr (\tilde{A},\tilde{B},\tilde{C},\tilde{D})$ is the probability density associated with $\tilde{A}$, $\tilde{B}$, $\tilde{C}$, $\tilde{D}$, while ∂_1_ is a control parameter for the joint structure *U*_*i*_*U*_*j*_ as

61 $${\partial }_{1}={C}^{\ast }(\tilde{A},\tilde{B},\tilde{C},\tilde{D}),$$

where ${C}^{\ast }(\tilde{A},\tilde{B},\tilde{C},\tilde{D})$ is an optimal control function using the post-processing results $\tilde{A}$, $\tilde{B}$, $\tilde{C}$, $\tilde{D}$, while ∂_2_ is a stabilization parameter associated with the stable gate *U*_*j*_, as

62 $${\partial }_{2}=\int \int \,{\rm{\Pr }}(\tilde{A},\tilde{B},\tilde{C},\tilde{D}){C}^{\ast }(\tilde{A},\tilde{B},\tilde{C},\tilde{D})d\tilde{A}d\tilde{B}={C}^{\ast }(\tilde{D}).$$

From (60), (61), and (62), C^*^(*U*_*i*_*U*_*j*_) is yielded as

63 $${C}^{\ast }({U}_{i}{U}_{j})={\partial }^{\ast }={C}^{\ast }(\tilde{A},\tilde{B},\tilde{C},\tilde{D}){C}^{\ast }(\tilde{D}),$$

such that the quantities of (61) and (62) are determined by ${\mathscr{C}}$ using the set of (59).

We also give the form of C^*^(*U*_*i*_*U*_*j*_) with respect to the cost function *f*_*C*_(*U*_*i*_*U*_*j*_). For this purpose, we also introduce time parameters for the controlling mechanism.

Let *T*_*L*_ be the time interval defined for the controlling as

64 $${T}_{L}=\,\mathrm{ln}\left(\frac{\sqrt{{\overline{A}}^{2}+{\overline{B}}^{2}}}{\sqrt{{\underline{A}}^{2}+{\underline{B}}^{2}}}\right)\frac{1}{-\vartheta },$$

where $\bar{x}$, $\underline{x}$ refer to the upper and lower bound on *x*, while *ϑ* is a decay rate parameter for the controlling, defined as

65 $$\vartheta ={\beta }_{i}^{0}-({\tilde{A}}^{2}+{\tilde{B}}^{2})-({\tilde{C}}^{2}+{\tilde{D}}^{2}).$$

For the activation of the ${\mathscr{C}}$ block, the A_*L*_ activation parameter is defined as

66 $$\begin{array}{rcl}{{\rm{A}}}_{L} & = & h((\sqrt{{\tilde{A}}^{2}+{\tilde{B}}^{2}})-(\sqrt{{\bar{A}}^{2}+{\bar{B}}^{2}}))\\  &  & -h((\sqrt{{\underline{A}}^{2}+{\underline{B}}^{2}})-(\sqrt{{\tilde{A}}^{2}+{\tilde{B}}^{2}}))\\  &  & +h((\sqrt{{\tilde{C}}^{2}+{\tilde{D}}^{2}})-{\beta }_{i}^{0}),\end{array}$$

where *h*(·) is the Heaviside function^115–117^ and ${\beta }_{i}^{0}$ is as shown in (22). As A_*L*_ > 0, the ${\mathscr{C}}$ block starts the calibration of the quantum gates; otherwise, there is no calibration in the system.

Then let *T*_*P*_ be the period time for one controlling cycle, defined as

67 $${T}_{P}={T}_{L}+{T}_{\bar{L}},$$

where ${T}_{\bar{L}}$ refers to an uncontrolled period, defined via ${\beta }_{i}^{0}$ as

68 $${T}_{\bar{L}}=\,\mathrm{ln}\left(\frac{\sqrt{{\overline{A}}^{2}+{\overline{B}}^{2}}}{\sqrt{{\underline{A}}^{2}+{\underline{B}}^{2}}}\right)\frac{1}{{\beta }_{i}^{0}}.$$

Let ∏ be the controlling amplitude and ${L}_{act}^{{\rm{\max }}}$ be maximal actuation level, as

69 $${L}_{act}^{{\rm{\max }}}=\frac{{\Pi }^{2}}{2}.$$

Using ∏ and a quasi-equilibrium assumption^115–117^, the parameter of (65) can be rewritten as

70 $$\vartheta ={\beta }_{i}^{0}-\ell {\Pi }^{2}={\beta }_{i}^{0}-\ell 2{L}_{act}^{{\rm{\max }}},$$

where *l* is defined as

71 $$\ell =\frac{({\tilde{A}}^{2}+{\tilde{B}}^{2})+({\tilde{C}}^{2}+{\tilde{D}}^{2})}{{\Pi }^{2}}.$$

The ${f}_{{\partial }^{\ast }}$ cost in function *f*_*C*_(*U*_*i*_*U*_*j*_) is therefore analogous to an ${\bar{L}}_{act}$ average actuation level at a particular $\tilde{A}$ and $\tilde{B}$, as

72 $${f}_{C}({U}_{i}{U}_{j})=({\tilde{A}}^{2}+{\tilde{B}}^{2})+\gamma {\bar{L}}_{act},$$

where *γ* is as in (42), while ${\bar{L}}_{act}$ is as

73 $$\begin{array}{rcl}{\bar{L}}_{act} & = & \left(\frac{{T}_{L}}{{T}_{P}}\right){L}_{act}^{max}=\left(\frac{{\beta }_{i}^{0}}{\ell {\Pi }^{2}}\right)\frac{{\Pi }^{2}}{2}\\  & = & \frac{{\beta }_{i}^{0}}{2\ell }=\frac{{\Pi }^{2}{\beta }_{i}^{0}}{2(({\tilde{A}}^{2}+{\tilde{B}}^{2})+({\tilde{C}}^{2}+{\tilde{D}}^{2}))}\mathrm{}.\end{array}$$

The optimal ∂^*^ = C^*^(*U*_*i*_*U*_*j*_) control function is therefore as

74 $$\begin{array}{rcl}{C}^{\ast }({U}_{i}{U}_{j}) & = & \mathop{{\rm{argmin}}}\limits_{\forall C({U}_{i}{U}_{j})}\,{f}_{C}({U}_{i}{U}_{j})\\  & = & \sqrt{{\bar{L}}_{act}}\\  & = & \sqrt{\frac{{\Pi }^{2}{\beta }_{i}^{0}}{2(({\tilde{A}}^{2}+{\tilde{B}}^{2})+({\tilde{C}}^{2}+{\tilde{D}}^{2}))}}\\  & = & {\partial }^{\ast },\end{array}$$

where *f*_*C*_(*U*_*i*_*U*_*j*_) is the cost function defined in (72).

The proof is concluded here. ■

### Direct L algorithm

Here we give the steps of the ${\mathscr{C}}$ controlling procedure assuming that there is no activation function (66) in the ${\mathscr{C}}$ block (referred to as Direct L Algorithm) and the *U*_*i*_*U*_*j*_ system is simplified as given by (31–36). The steps are summarized in Algorithm A.1.

#### **Algorithm 1** Direct L Algorithm

**Figure:**
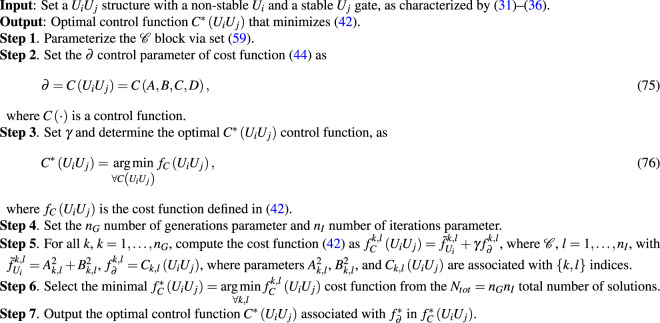

### Controlling on the quantum bus

#### **Lemma 2**

*The quantum states placed onto the qubus can be calibrated by operation U*_*C*_.

**Proof**. The proof assumes that the aim of *U*_*C*_ is the correction of the qubus states before the *M*_*b*_ measurement is performed.

The $|{\varphi }_{i}\rangle$, *i* = 1, …, *n* quantum states placed onto the qubus are transformed into the reference state by the ${U}_{C}=\{{U}_{C}^{1},\ldots ,{U}_{C}^{n}\}$ operation (qubus operation), where ${U}_{C}^{i}$ is the operation associated with the *i*-th qubit. The controlling is as follows.

Since for a particular $|{\varphi }_{i}\rangle$, the difference in the reference phase space angle ±*θ*_*i*_ of an *i*-th gate *U*_*i*_ can be identified by (10) and (11) via the *M* measurement and *P* post-processing, for Δ_*i*_ ≠ 0, a correction operator ${U}_{C}^{i,{\varDelta }_{i}}$ can be straightforwardly defined as

77 $${U}_{C}^{i,{\Delta }_{i}}={e}^{i\varsigma }{R}_{C}^{i,{\Delta }_{i}}({\eta }_{i}),$$

where $\varsigma$ is a real number, and ${R}_{C}^{i,{\Delta }_{i}}({\eta }_{i})$ is as

78 $${R}_{C}^{i,{\Delta }_{i}}({\eta }_{i})=\,\cos \left(\frac{{\eta }_{i}}{2}\right)I-i\,\sin \left(\frac{{\eta }_{i}}{2}\right)\bar{n}\cdot \overrightarrow{\sigma }=\exp \left(-i\frac{{\eta }_{i}}{2}\bar{n}\cdot \overrightarrow{\sigma }\right),$$

where $\bar{n}$ is a unit vector as $\bar{n}=({n}_{x},{n}_{y},{n}_{z})=(a/L,b/L,c/L)$, where *a*, *b*, *c* and ${\mathscr{C}}$ are real numbers determined by the ${H}_{C}^{i,{\Delta }_{i}}$ correction Hamiltonian ${H}_{C}^{i,{\Delta }_{i}}$, as

79 $${H}_{C}^{i,{\Delta }_{i}}=a{\sigma }_{X}+b{\sigma }_{Y}+c{\sigma }_{Z}=L\bar{n}\cdot \overrightarrow{\sigma },$$

where $L=\sqrt{{a}^{2}+{b}^{2}+{c}^{2}}$, while $\overrightarrow{\sigma }=({\sigma }_{X},{\sigma }_{Y},{\sigma }_{Z})$ is the Pauli vector, ${(\bar{n}\cdot \overrightarrow{\sigma })}^{2}=I$, and

80 $${\eta }_{i}=\{\begin{array}{l}|{\varsigma }_{i}{\phi }_{i}|,\,{\rm{if}}\,{\theta {\prime} }_{i} < {\theta }_{i}\\ 2\pi -|{\varsigma }_{i}{\phi }_{i}|,\,{\rm{if}}\,{\theta {\prime} }_{i} > {\theta }_{i}\end{array},$$

where *ϕ*_*i*_ is given in (53).

For *λ*_*i*_ ≠ 0, a correction operator ${U}_{C}^{i,{\lambda }_{i}}$ can be straightforwardly defined as

81 $${U}_{C}^{i,{\lambda }_{i}}={e}^{i\varsigma }{R}_{C}^{i,{\lambda }_{i}}({\nu }_{i}),$$

where

82 $${R}_{C}^{i,{\lambda }_{i}}({\nu }_{i})=\,\cos \,\frac{{\nu }_{i}}{2}I-i\,\sin \,\frac{{\nu }_{i}}{2}\bar{n}\cdot \overrightarrow{\sigma }=\exp \left(-i\frac{{\nu }_{i}}{2}\bar{n}\cdot \overrightarrow{\sigma }\right),$$

where

83 $${\nu }_{i}=\{\begin{array}{l}2\pi -|{\varsigma }_{i}{\varphi }_{i}|,\,{\rm{if}}\,-{\theta {\prime} }_{i} > -{\theta }_{i}\\ |{\varsigma }_{i}{\varphi }_{i}|,\,{\rm{if}}\,-{\theta {\prime} }_{i} < -{\theta }_{i}\end{array},$$

where ${\varphi }_{i}$ is given in (57), and with the corresponding *a*, *b*, *c* and ${\mathscr{C}}$ parameters of the Hamiltonian ${H}_{C}^{i,{\lambda }_{i}}$. The operators (77) and (82) therefore calibrate the qubit onto the reference position $|{\varphi }_{i}\rangle ={U}_{i}|0\rangle$ or $|{\varphi }_{i}\rangle ={U}_{i}|1\rangle$ associated with the reference angle ±*θ*_*i*_ via Δ_*i*_ (10) and *λ*_*i*_ (11), yielded by the *M* measurement. If Δ_*i*_ = 0 or *λ*_*i*_ = 0, then $|{\varphi }_{i}\rangle$ is in the correct position; therefore, ${U}_{C}^{i}=I$; thus, the final form of ${U}_{C}^{i}$ is

84 $${U}_{C}^{i}=\{\begin{array}{l}{U}_{C}^{i,{\Delta }_{i}},\,{\rm{if}}\,{\Delta }_{i}\ne 0\\ {U}_{C}^{i,{\lambda }_{i}},\,{\rm{if}}\,{\lambda }_{i}\ne 0\\ I,\,{\rm{if}}\,{\Delta }_{i}=0\,{\rm{or}}\,{\lambda }_{i}=0\end{array}.$$

The proof is therefore concluded here. ■

## Conclusions

Here, we defined a method for unsupervised control of entangled quantum gates in gate-model quantum computers and near-term quantum devices. The framework utilizes the terms of control theory for the description of the control problem. The system model uses the quantum bus scheme that correlates the gate outputs with auxiliary probe beams. The probe states are measured to provide information to a post-processing unit and are then inputted into the machine learning control. The entangled gate structure is achieved by a second measurement block that entangles the gate outputs, leading to an entangled gate structure. We showed that if the quantum gates are entangled, the non-stable gate is controllable by a stable quantum gate; however, if the gates formulate a product system, then the random oscillations of the non-stable gate are not controllable in terms of control theory. The solution stabilizes the quantum gate structure via the derivation of an optimal control function that minimizes a particular cost function. The framework provides an implementable solution for experimental quantum computations and for near-term quantum computer architectures in the quantum Internet.

### Submission note

Parts of this work were presented in conference proceedings^47^.

### Ethics statement

This work did not involve any active collection of human data.

## Supplementary information

Supplemental Information.
