# Supplementary material for: Unsupervised Quantum Gate Control for Gate-Model Quantum Computers
Source: Sci Rep. 2020 Jul 1;10:10701. doi: 10.1038/s41598-020-67018-1 (PMC7329862; doi:10.1038/s41598-020-67018-1)
Supplement: Supplementary file 1 — Supplemental Information. [file 41598_2020_67018_MOESM1_ESM.pdf]

Unsupervised Quantum Gate Control for Gate-Model Quantum Computers

Laszlo Gyongyosi[1,2,3,*]

[1]School of Electronics and Computer Science, University of Southampton, Southampton, SO17 1BJ, UK
[2]Department of Networked Systems and Services, Budapest University of Technology and Economics, Budapest, H-1117 Hungary
[3]MTA-BME Information Systems Research Group, Hungarian Academy of Sciences, Budapest, H-1051 Hungary
[*]gyongyosi@hit.bme.hu

ABSTRACT

In near-term quantum computers, the operations are realized by unitary quantum gates. The precise and stable working mechanism of quantum gates is essential for the implementation of any complex quantum computations. Here, we define a method for the unsupervised control of quantum gates in near-term quantum computers. We model a scenario in which a tensor product structure of non-stable quantum gates is not controllable in terms of control theory. We prove that the non-stable quantum gate becomes controllable via a machine learning method if the quantum gates formulate an entangled gate structure.

A Appendix

A.1 Abbreviations

NISQ Noisy Intermediate-Scale Quantum

NMR Nuclear Magnetic Resonance

A.2 Notations

The notations of the manuscript are summarized in Table A.1.

Table A.1. Summary of notations.

Notation	*Description*
U_i	An i-th unitary gate. It also refers to the unitary operator achieved by the i-th unitary gate, as $U_i = \begin{cases} U_i, \text{ if } \Delta_i = 0 \text{ or } \lambda_i = 0 \\ V_i, \text{ if } \Delta_i \neq 0 \\ Q_i, \text{ if } \lambda_i \neq 0 \end{cases}$, where Δ_i and λ_i are some real parameters.
U_iU_j	Entangled structure (joint system) of quantum gates U_i and U_j.
$U_i \otimes U_j$	Product system of gates U_i and U_j.
H_{U_i}	Hamiltonian of U_i.
T_i	Period time associated to Hamiltonian H_{U_i} of U_i, $T_i = zt_{U_i}$, where $z > 0$.
t_{U_i}	Application time of unitary U_i.
E_i	Energy associated to Hamiltonian H_{U_i}, $E_i = \frac{1}{2}\hbar 2\pi (f_i)$, where f_i is the frequency $f_i = \frac{1}{T_i}$, where T_i is the period time.
n	Total number of unitary gates.

$\|\varphi_i\rangle$	Output of gate U_i.
H	Hamiltonian operator.
Q_b	Quantum bus.
d	Dimension of the quantum system.
$\|0\rangle_i$	Auxiliary qubit system, probe beam (a continuous quantum variable).
θ_i	Reference angle in the phase space associated to unitary gate U_i.
M	Homodyne measurement on the auxiliary systems.
M_b	Homodyne measurement for the creation of the entangled structure.
P	Post processing unit.
$\mathscr{C}$	Machine learning control block.
U_C	Block for the correction of the actual $\|\varphi_i\rangle$ states on the quantum bus Q_b.
$C(\cdot)$	Control function.
$C^*(\cdot)$	Optimal control function
M	Vector of measurement results.
∂	Control parameter.
s	System state, $s = P(M)$.
f_C	Cost function subject to a minimization.
f_s	Cost function associated to s.
f_∂	Cost function associated to control parameter ∂.
H_{int}^i	Interaction Hamiltonian, $H_{int}^i = \hbar\chi_i\sigma_Z a^\dagger a$, where χ_i is the strength of the nonlinear interaction, a and $a^\dagger$ are the creation and annihilation operators, while σ_Z is the Pauli Z-operator.
t_{int}^i	Interaction time for an i-th probe beam $\|0\rangle_i$.
V_i,Q_i	Unitary operators, $V_i \neq U_i$, and $Q_i \neq U_i$.
θ_i', $-\theta_i'$	Phase shift at $V_i \neq U_i$ and $Q_i \neq U_i$, expressed as $\theta_i' = \theta_i + \Delta_i$, and $-\theta_i' = -(\theta_i + \lambda_i)$.
$\|\tilde{0}\rangle_i$	State of the probe beam state after the interaction.
$\|0\rangle_b$	Probe beam for the creation of the entangled structure.
$M(\|\tilde{0}\rangle_i)$	Measurement of $\|\tilde{0}\rangle_i$.
$M_b(\|0\rangle_b)$	Measurement of $\|0\rangle_b$.
Δ_i	Noise term, $\Delta_i = \cos^{-1}\left(\frac{M(\|\tilde{0}\rangle_i)}{x}\right) - \theta_i$, where x and θ_i are known parameters.
λ_i	Noise term, $\lambda_i = -\left(\cos^{-1}\left(\frac{M(\|\tilde{0}\rangle_i)}{x}\right) + \theta_i\right)$, where x and θ_i are known parameters.
t_b	Interaction time with probe beam $\|0\rangle_b$.
$\pm\omega_i^b$	Rotation angle of $\|0\rangle_b$ for a $\|\varphi_i\rangle$.
A, B	Parameters defined for U_i, as $A = \cos(t_{U_i})$, $B = \sin(t_{U_i})$, where $t_{U_i}$is the application time of U_i.

C, D	Parameters defined for U_j, as $C = \cos\left(t_{U_j}\right)$, $D = \sin\left(t_{U_j}\right)$, where $t_{U_j}$is the application time of U_j.
μ_i	Growth rate parameter defined for U_i.
β_i^i	Initial growth rate parameter for U_i.
β_i^i	Parameter for growth-rate change of μ_i due to $\sqrt{(A^2+B^2)}$, defined for U_i.
β_i^j	Parameter for growth-rate change of μ_i due to $\sqrt{(C^2+D^2)}$, defined for U_i.
$\mathscr{C}_{U_i}$, $\mathscr{C}_{U_j}$	Sets, defined for U_i and U_j as $\mathscr{C}_{U_i} : \{D_t(A), D_t(B)\}$, and $\mathscr{C}_{U_j} : \{D_t(C), D_t(D)\}$, where $D_t(x) = dx/dt$.
δ_i^i	Parameter for frequency-change due to term $\sqrt{(A^2+B^2)}$, defined for U_i.
δ_i^j	Parameter for frequency-change due to term $\sqrt{(C^2+D^2)}$, defined for U_i.
f_i	Initial frequency parameter, $f_i = \frac{1}{T_i}$, where T_i is the period time.
F_i	Parameter for the frequency of U_i defined as $F_i = f_i + \delta_i^i\left(A^2+B^2\right) + \delta_i^j\left(C^2+D^2\right)$.
$\tilde{f}_{U_i}$	Cost function for the calibration of the non-stable U_i in U_iU_j, defined as $\tilde{f}_{U_i} = A^2+B^2$.
f_∂	Cost function associated to control parameter ∂, as $f_\partial = \partial^2$.
γ	Penalization parameter.
$f_C(U_iU_j)$	Cost function associated to the control of the entangled structure U_iU_j, $f_C(U_iU_j) = \tilde{f}_{U_i} + \gamma f_\partial$.
$C(U_iU_j)$	Control function defined for the joint structure U_iU_j.
$\Pr(A,B,C,D)$	Probability density associated to a current values of A,B,C,D.
∂_{U_i}	Expectation value of ∂ on U_i at a particular A,B of U_i, $\partial_{U_i} = \mathbb{E}\left(C(U_iU_j)\middle\vert A,B\right)$.
∂_{U_j}	Expectation value of ∂ on U_j at a particular C,D of U_j, $\partial_{U_j} = \mathbb{E}\left(C(U_iU_j)\middle\vert C,D\right)$.
$\tilde{A}$,$\tilde{B}$, $\tilde{C}$, $\tilde{D}$	Estimates of A,B, C,D
ς_i	Parameter, defined as $\varsigma_i = t_{U_i}/\theta_i$.
ϕ_i	Phase space angle.
t_{ϕ_i}	Time parameter associated to ϕ_i.
φ_i	Phase space angle.
t_{φ_i}	Time parameter associated to φ_i.
$\bar{L}_{act}$	Average actuation level.
$\mathscr{S}_f$	Set of operations, $\mathscr{S}_f : \{S_e, S_t\}$, where S_e is a set of elementary operations $S_e = \{\pm, \times, /\}$, while $S_t = \{\exp, \sin, \ln, \tanh\}$ is a set of transcendental functions.
Π	Controlling amplitude.
∂^*	Optimal control parameter of the for the joint structure U_iU_j.
T_L	Time interval defined for the controlling.
ϑ	Decay rate parameter for the controlling.
A_L	Activation parameter, if $\mathrm{A}_L > 0$, the $\mathscr{C}$ blocks starts the calibration of the quantum gates, otherwise there is no any calibration in the system.

$h(\cdot)$	Heaviside function.
T_P	Period time for one controlling cycle.
$T_{\overline{L}}$	Uncontrolled period.
$L_{act}^{\max}$	Maximal actuation level, $L_{act}^{\max} = \frac{\Pi^2}{2}$.
ℓ	Parameter, defined as $\ell = \frac{\left(\tilde{A}^2+\tilde{B}^2\right)+\left(\tilde{C}^2+\tilde{D}^2\right)}{\Pi^2}$.
U_C^{i,Δ_i}	Correction operator for $\Delta_i \neq 0$.
$R_C^{i,\Delta_i}(\eta_i)$	Rotation operator for U_C^{i,Δ_i}, where η_i is an angle.
H_C^{i,Δ_i}	Correction Hamiltonian H_C^{i,Δ_i}, defined as $H_C^{i,\Delta_i} = a\sigma_X + b\sigma_Y + c\sigma_Z = L\vec{n}\cdot\vec{\sigma}$, where $L = \sqrt{a^2+b^2+c^2}$, while $\vec{\sigma} = (\sigma_X, \sigma_Y, \sigma_Z)$ is the Pauli vector.
U_C^{i,λ_i}	Correction operator for $\lambda_i \neq 0$.
$R_C^{i,\lambda_i}(\nu_i)$	Rotation operator for U_C^{i,λ_i}, where ν_i is an angle.
ς	Real number.
I	Identity operator.
U_C^i	Correction operator for the i-th state $\lvert\varphi_i\rangle$ on the qubus.
